# Changes in Substance Use Treatment Providers’ Delivery of the 5A’s for Non-Cigarette Tobacco Use in the Context of a Comprehensive Tobacco-Free Workplace Program Implementation

**DOI:** 10.3390/ijerph20032730

**Published:** 2023-02-03

**Authors:** Ammar D. Siddiqi, Tzuan A. Chen, Maggie Britton, Isabel Martinez Leal, Brian J. Carter, Virmarie Correa-Fernández, Anastasia Rogova, Bryce Kyburz, Teresa Williams, Kathleen Casey, Lorraine R. Reitzel

**Affiliations:** 1 Department of Health Disparities Research, The University of Texas MD Anderson Cancer Center, 1400 Pressler St., Houston, TX 77030, USA; 2 Department of Biosciences, Rice University, 6100 Main St., Houston, TX 77005, USA; 3 Department of Psychological, Health & Learning Sciences, The University of Houston, 3657 Cullen Blvd Stephen Power Farish Hall, Houston, TX 77204, USA; 4 HEALTH Research Institute, The University of Houston, 4349 Martin Luther King Blvd., Houston, TX 77204, USA; 5 Integral Care, 1430 Collier St., Austin, TX 78704, USA

**Keywords:** tobacco control, behavioral health, provider self-efficacy, non-cigarette tobacco use, brief intervention, tobacco use cessation, workplace program, barriers, substance use disorder, provider training

## Abstract

Tobacco use treatment is not prioritized in substance use treatment centers (SUTCs), leading to tobacco-related health inequities for patients with substance use disorders (SUDs) and necessitating efforts to enhance providers’ care provision. Training providers on how to treat tobacco use increases their intervention on patients’ smoking, but limited work addresses its effects on their non-cigarette tobacco use intervention provision. This study redressed this gap using data from 15 unaffiliated SUTCs in Texas (serving 82,927 patients/year) participating in a tobacco-free workplace program (TFWP) that included provider education on treating tobacco use, including non-cigarette tobacco use. SUTC providers completed surveys before (n = 259) and after (n = 194) TFWP implementation. Past-month screening/intervention provision for non-cigarette tobacco use (the 5A’s; ask, advise, assess, assist, arrange) and provider factors theoretically and practically presumed to underlie change [i.e., beliefs about concurrently treating tobacco use disorder (TUD) and other SUDs, self-efficacy for tobacco use assessment (TUA) delivery, barriers to treating tobacco dependence, receipt of tobacco intervention training] were assessed. Generalized linear or linear mixed models assessed changes over time from before to after TFWP implementation; low vs. high SUTC-level changes in provider factors were examined as moderators of changes in 5A’s delivery. Results indicated significant improvement in each provider factor and increases in providers’ asking, assisting, and arranging for non-cigarette tobacco use over time (*p*s < 0.04). Relative to their counterparts, SUTCs with high changes in providers’ beliefs in favor of treating patients’ tobacco use had greater odds of advising, assessing, assisting, and arranging patients, and SUTCs with greater barrier reductions had greater odds of advising and assisting patients. Results suggest that TFWPs can address training deficits and alter providers’ beliefs about treating non-tobacco TUD during SUD care, improve their TUA delivery self-efficacy, and reduce intervention barriers, ultimately increasing intervention provision for patients’ non-cigarette tobacco use. SUTCs with the greatest room for improvement in provider beliefs and barriers to care provision seem excellent candidates for TFWP implementation aimed at increasing non-cigarette tobacco use care delivery.

## 1. Introduction

Tobacco use has been attributed to causing several diseases including but not limited to cancer, diabetes, respiratory diseases, and heart disease [1]. As such, it has been recognized as the leading cause of preventable illness and disease in the U.S., being responsible for nearly 480,000 deaths annually. The majority of this tobacco-related mortality is caused by smoking conventional cigarettes, which have over a billion global users and are the most popular type of tobacco product by far [2]. Despite that rates of cigarette smoking have been on the decline in recent decades in the U.S., the success of tobacco control efforts has been impeded by the diversification of tobacco products [3,4,5,6]. Recent research has demonstrated that the concurrent use of multiple types of non-cigarette tobacco products (i.e., cigars, smokeless tobacco, snuff, hookah, e-cigarettes, and pipes) is becoming increasingly common [7,8,9,10]. The increased variety of tobacco products in the commercial marketplace has been met with an alarming surge in demand that threatens decades of successful tobacco control efforts [4,5,11,12,13].

Among populations that have been most impacted by the development of non-cigarette tobacco products are individuals suffering from non-tobacco substance abuse disorders, who are more likely to be diagnosed with mental illness and reportedly use non-cigarette tobacco products up to rates 10 times greater than that of the general population [14,15,16,17]. Concerningly, the use of non-cigarette tobacco products has been associated with an increased probability of cigarette smoking initiation and co-occurring use of other addictive drugs [18,19,20,21]. Moreover, for individuals already struggling with mental illness or substance use disorders, the use of additional drugs can exacerbate current symptoms or introduce a slew of additional health problems [22,23]. This group also experiences limited access to cessation care, reduced rates of tobacco screenings, and targeted advertisements from the tobacco industry, making poorer health outcomes nearly inevitable [24,25,26,27]. The disproportionate use of non-cigarette tobacco products among substance use disorder populations and concomitant health effects reinforce their status as a tobacco disparity group [28].

Substance use treatment centers (SUTCs) represent an ideal setting for tobacco-using patients to be advised on how to quit using tobacco products. However, tobacco use screenings, a natural first step prior to the delivery of evidence-based interventions, are not pervasive within SUTCs. A recent Texas-based study published in 2022 indicated that 80% of participating SUTCs mandated tobacco use screenings, an increase from 70.2% recorded in a 2016 study, though samples may have differed between studies [29,30]. While these statistics potentially indicate an improvement in SUTC tobacco screening delivery over time, sizeable gaps in screening practice still exist with the growing spectrum of tobacco products. Research suggests that screening for non-cigarette tobacco products occurs as infrequently as one-fourth of the time that cigarette smoking is queried [31,32,33,34,35]. As a result, providers may not be aware of potential non-cigarette tobacco use and are therefore unlikely to offer advice or counseling. These practices are not in line with the most recent clinical guidelines that encourage every provider to screen for all tobacco use and provide brief evidence-based intervention consisting of the 5A’s (asking patients about their tobacco use, advising them to quit, assessing their willingness to quit, assisting them in quitting, and arranging for follow-up) at every patient encounter when any tobacco use is endorsed [36]. 

Despite the efficacy of these guidelines for treating tobacco dependence, provider barriers exist that prevent their application in clinical practice. Providers have frequently reported difficulty in providing tobacco cessation care as a result of a lack of knowledge, time, and available resources; perceived irrelevance to the chief complaint; and low confidence in patient compliance [29]. The array of barriers experienced and the wide-reaching influence that providers have on patient behavior highlight missed opportunities to reduce tobacco use among their patients. One way to bolster the provision of non-cigarette tobacco care delivery is through provider education. Training provision as a part of comprehensive tobacco-free workplace programs (i.e., multicomponent programs that include tobacco-free workplace policies, education, specialized training, etc.; for examples see [37,38]) has been recognized for its brief yet impactful nature in improving provider smoking cessation practices [37,39,40,41,42,43,44,45]. Moreover, observable increases in provider tobacco care knowledge, motivation to intervene, ability to surmount barriers, self-efficacy, and positive outlook on treating tobacco dependence have been found after training periods as short as one hour [46,47]. Although most literature is focused on the effects of provider training on cigarette smoking intervention provision, education may be equally critical to non-cigarette tobacco use intervention. A recent study found that providers who believed patients were concerned about their non-cigarette tobacco use thought it was important to offer non-cigarette tobacco use cessation counseling, felt they were equipped with the skills to do so, and were familiar with referral options, were more likely to deliver the 5A’s to patients with behavioral health needs [48]. These results highlight several factors that can be targeted in provider training; however, longitudinal research is needed to support SUTC workplace interventions that can improve these metrics and ultimately impact providers’ non-cigarette tobacco intervention practices. 

The current study was designed to address gaps in the literature by delineating the impact of a comprehensive tobacco-free workplace intervention (that included provider education) on the delivery of the 5A’s for non-cigarette tobacco use within participating SUTCs in Texas. Based on the social cognitive theory of behavior change [49], provider education and other intervention program components were designed to positively affect provider beliefs about intervening in tobacco use, provider self-efficacy for tobacco use assessment (TUA) conduct, the receipt of recent intervention training, and perceived treatment barriers, thereby increasing the provision of tobacco use care to their patients. Herein, changes in provider factors (i.e., beliefs, self-efficacy, training receipt, and barriers) and their use of the 5A’s for non-cigarette tobacco use were examined from pre- to post-program implementation. Additionally, the effects of high versus low changes in provider factors at the SUTC level were examined relative to changes in the delivery of 5A’s for non-cigarette tobacco use over time. In addressing these multiple research gaps, this work expands the literature regarding how a comprehensive tobacco-free workplace program implementation may affect care for patients with non-cigarette tobacco use (an understudied area) within SUTCs (a real-world setting where many such patients receive care) while understanding more about how provider factors within the center may shift during implementation and how those shifts may moderate provider intervention practice changes. Ultimately, results may inform implementation science designed to reduce the research-to-practice gap in tobacco control by understanding how to increase SUTCs’ capacity to screen for and treat their patients’ non-cigarette tobacco use with evidence-based interventions. 

## 2. Materials and Methods

### 2.1. Participants and Recruitment

Participants were providers from 15 (of 19) SUTCs in Texas who enrolled in and completed Taking Texas Tobacco Free (TTTF), a comprehensive tobacco-free workplace program detailed in several prior publications [37,40,41,42,43]. Recruitment of centers entailed direct email solicitation of known SUTCs, promotion at professional conferences for substance use treatment providers, and word of mouth. Enrollment in the program was initiated by the leadership of each center and was ongoing from December 2017 to May 2020. More information on the 4 SUTC withdrawals is available in other work [42]. The participating SUTCs served in excess of 80,000 patients yearly through approximately 300,000 contacts, with coverage across 9 counties in Texas (Harris, Victoria, Bexar, Nueces, Travis, Grayson, Tarrant, Dallas, and Galveston County). Some SUTCs served unique populations such as sexual and gender minorities and women and families; more details about participating SUTCs are available in prior work [42]. Participation in TTTF spanned 7.2 to 13.6 (10.96 ± 3.84) months, during which SUTCs implemented each component of the program, with duration varying based on center capacity, competing demands, staff availability, etc. 

### 2.2. Program Implementation

The University of Houston’s Institutional Review Board approved study procedures. TTTF implementation entailed a 1- to 2-h education session for all staff (providers and non-patient-facing employees). This educational session reviewed the health risks of tobacco use for patients with substance dependencies, the importance and beneficial outcomes of treating tobacco use within substance use dependency treatment settings, and medications and counseling interventions to assist with tobacco use disorder (TUD). Specifically, the use of the 5A’s intervention for conventional cigarette smoking and, as relevant to this report, non-cigarette tobacco use was reviewed and promoted as an intervention that should occur at every patient contact. The 5A’s entailed asking the patient if they use non-cigarette tobacco products, advising them to quit, assessing their interest in quitting, assisting in a quit attempt through intervention or referral, and arranging a follow-up to discuss quit progress or renew interest in making a quit attempt. This educational session was delivered by trained TTTF staff who were behavioral health treatment providers themselves, with duration and delivery (in person or virtual) negotiated with each center’s leadership. Another aspect of the TTTF intervention was to send program champions (i.e., providers at each center whose leadership tasked them with co-leading the implementation partnership with the TTTF team) to a multi-day certified tobacco treatment specialist training delivered by an accredited program. Program champions did not receive additional financial compensation for taking on this position. All center providers were also invited to attend a 1-day motivational interviewing training delivered by experts on the TTTF team, with 78 providers choosing to attend. Starter kits of NRT were also provided by TTTF for patients and staff at each enrolled center and they were encouraged and assisted to budget for its continued availability over time. Distribution of NRT was based upon the size of the SUTC, their patients’ and employees’ tobacco use rates, and the SUTC’s capacity for NRT storage and distribution on-site; the amount was decided in collaboration with the SUTC and, in many cases, entailed multiple shipments (range = 1–4 per SUTC). Participating SUTCs each received between 48 and 672 boxes of NRT gum, patches, and lozenges of 6299 boxes distributed (total value = $137,393.61). Additionally, each participating center implemented a comprehensive tobacco-free workplace policy that disallowed all tobacco product use indoors or on the premises. TTTF worked with leadership on policy wording, quality assurance measures, and enforcement procedures, while providing permanent signage about the policy for the grounds. The last component of TTTF was the design and provision of health promotion materials (e.g., brochures about the danger of tobacco use and benefits of quitting) that could be given to patients and posters for treatment rooms advising patients to talk with their healthcare provider about their tobacco use. Feedback on dissemination materials was solicited from center staff to tailor images to fit the demographics and needs of those they served; materials focused on conventional cigarette use as well as non-cigarette tobacco product use, including smokeless tobacco, e-cigarettes, etc. 

### 2.3. Survey Procedures 

Data were collected from providers (i.e., those with direct patient contact, e.g., counselors, prescribers, and social workers) at each participating center via an electronic survey that was distributed by the program champion at the request of TTTF staff pre- and post-implementation of the program components. Surveys had a cover letter describing the voluntary nature of data collection and its anonymity, which was designed to maintain privacy and encourage honest responses given that the survey assessed the providers’ treatment practices. The number of providers completing pre-implementation surveys within centers ranged from 3 to 65 (total pre-implementation n = 259 for all centers combined). The number of providers completing post-implementation surveys within centers ranged from 1 to 50 (total post-implementation n = 194 for all centers combined). The response rate cannot be calculated as the exact number of providers at each center was not collected; however, data collected at pre-implementation indicated that 10 of the 15 centers employed <50 providers. Anecdotally, some centers had as few as 5 providers. High rates of provider turnover at SUTCs during the implementation process were expected [50,51,52,53]; therefore, it is likely that some proportion of providers participated in only the pre- or post-implementation survey, whereas some proportion participated in both data collections. Based on the anonymous nature of data collection, pre- and post-implementation surveys could be matched only at the SUTC level and not at the provider level. Each survey distribution lasted approximately 3 weeks until closure; completion reminders were sent to providers regularly during this period. 

### 2.4. Survey Measures

Each of the following items was assessed in both the pre- and post-implementation surveys: beliefs about the importance of providing tobacco use disorder (TUD) care, their self-efficacy for delivering TUAs, their receipt of recent intervention training to treat tobacco use, perceived barriers to providing TUD care, and delivery of each of the 5A’s for non-cigarette tobacco use. Each variable was analyzed as binary, as described further below. This classification was consistent with prior work examining provider factors [48] and provider delivery of the 5A’s for cigarette smoking [39,40,41,54,55] and for non-cigarette tobacco use [48], enhancing comparability with extant publications. 

**Provider beliefs.** Provider beliefs were assessed with an item reading: “It is as critical to treat tobacco use as it is to treat other substance use throughout treatment.” Response options assessed the level of agreement with the statement on a 5-point Likert scale ranging from “strongly agree” to “strongly disagree.” A binary variable was created for analysis (1 = strongly agree and somewhat agree vs. 0 = neither agree nor disagree, somewhat disagree, and strongly disagree). 

**Provider self-efficacy.** Provider self-efficacy was assessed with an item reading: “I feel like I am able to effectively deliver TUAs to patients.” Response options assessed the level of agreement with the statement on a 5-point Likert scale ranging from “strongly agree” to “strongly disagree.” A binary variable was created for analysis (1 = strongly agree and somewhat agree vs. 0 = neither agree nor disagree, somewhat disagree, and strongly disagree). 

**Receipt of intervention training.** Providers’ receipt of intervention training was assessed with an item reading: “In the last 12 months, have you received any training on the use of counseling and behavioral therapies to treat tobacco use (e.g., motivational interviewing)?” Response options were 0 = no or 1 = yes. 

**Perceived barriers to treatment.** Providers were asked: “What barriers do you face in regularly treating tobacco-using patients?” Providers could select as many of the following that applied: lack of knowledge on (1) how to treat tobacco with medications, or (2) with counseling; (3) where to refer patients for assistance; (4) how to motivate a quit attempt; beliefs that (5) tobacco-using patients do not want to quit or (6) cannot quit; that the provider (7) did not have time to treat tobacco use, or (8) that the center did not want them to treat tobacco use. Responses to this item were summed for a total score (number of perceived barriers to TUD care). 

**Providers’ delivery of the 5A’s for non-cigarette tobacco use.** Providers were asked: “In your clinical work here last month, did you ask patients about their use of other tobacco products besides conventional cigarettes?” For those who answered yes, the remaining four items used the common prompt: “With regard to patients you saw last month who used other tobacco products, did you …” and respectively queried if they advised them to quit, assessed their willingness to make a quit attempt, assisted them to quit by providing treatment or making a referral for treatment, and arranged to follow up with them to assess their progress regarding quitting. Response options for each of these items were “yes, all of them”, “yes, some of them”, and “no, none of them.” For analytic purposes, responses to each query were coded as no = 0 (“no, none of them”) or yes = 1 (“yes, all of them” and “yes, some of them”). This approach is consistent with several prior studies in the area focusing on the 5A’s for cigarette use (see [35,37,39,41,54,55,56]) and for non-cigarette use (see [35,54]), facilitating comparison with extant work. 

### 2.5. Statistical Analysis

Statistical analyses were conducted in three steps. First, the effect of tobacco-free workplace program implementation on providers’ provision of the 5A’s for non-cigarette tobacco use from pre- to post-implementation was investigated. Second, the effect of tobacco-free workplace program implementation on provider beliefs, provider self-efficacy, receipt of intervention training, and perceived barriers to treatment over time was examined. Third, whether the change of 5A’s delivery for non-cigarette tobacco use over time differed by low versus high center-level changes (i.e., moderation analyses) in provider beliefs, provider self-efficacy, receipt of intervention training, and/or perceived barriers to treatment was assessed. The moderation effects were examined by including the interaction terms of time and binary center-level changes in the models. 

Due to unmatched data at the provider (i.e., individual) level, independent variables (i.e., provider beliefs, provider self-efficacy, receipt of intervention training, and perceived barriers) were aggregated to the SUTC/center level for pre- and post-implementation surveys. Changes in the center-level independent variables (i.e., %/mean at post-implementation—%/mean at pre-implementation) were calculated for each center. Binary (derived from median split) center-level independent variables were created for moderation analyses, representing high (=1) vs. low (=0) changes over time. Consequently, resulting variables represented, for example, greater changes in provider beliefs that tobacco use should be addressed during substance use treatment over time [vs. smaller changes], greater changes in the number of barriers to routinely providing tobacco use disorder care [vs. smaller changes], and so on.

The nested data structure of providers (level 1) within the SUTCs (level 2) was accounted for through generalized linear mixed models (generalized linear mixed model, binomial distribution, logit link, and variance components for the variance matrix) in steps 1 and 3 of the analyses; linear mixed models were used in step 2. Four centers were excluded from analyses involving provider self-efficacy for tobacco use screening, as this item was not administered to centers enrolled at an early phase of this study. All analyses were conducted using SAS Version 9.4 and the level of significance was designated at *p* < 0.05.

## 3. Results

### 3.1. Substance Use Center Characteristics

At pre-implementation, a center-level average of 82.33% (SD = 13.21) of SUTC providers believed that tobacco use should be addressed during substance use treatment, 67.98% (SD = 21.44) reported self-efficacy for the delivery of TUAs, and 39.06% (SD = 12.70) reported receiving recent intervention training. Overall, a center-level average of 1.60 (SD = 0.67) barriers to routinely providing TUD care were endorsed by SUTC providers. By post-implementation, mean center-level beliefs that tobacco use should be addressed during substance use treatment increased by 6.78% (SD = 19.85), self-efficacy for the delivery of TUAs increased by 4.14% (SD = 21.16), and recent receipt of intervention training increased by 35.68% (SD = 25.23). Alternatively, the number of barriers to routinely providing TUD care at the center level decreased by an average of 0.73 (SD = 0.76). 

### 3.2. The Effect of Comprehensive Tobacco-Free Workplace Implementation on the 5A’s for Non-Cigarette Tobacco Use

At pre-implementation, 50.97% of providers asked patients about their non-cigarette tobacco use, 56.72% advised them to quit, 67.67% assessed their willingness to make a quit attempt, 48.87% assisted with quitting through intervention or referral, and 39.10% arranged a follow-up. At post-implementation, 64.43% of providers asked patients about their non-cigarette tobacco use, 60.12% advised them to quit, 65.34% assessed their willingness to make a quit attempt, 58.62% assisted with quitting through intervention or referral, and 53.45% arranged a follow-up. See Table 1 for providers’ delivery of the 5A’s for non-cigarette tobacco use at pre- and post-program implementation by SUTC.

Results from generalized linear mixed models assessing changes from pre- to post-implementation showed significant increases in providers asking patients about their use of non-cigarette tobacco products (Estimate = 0.608, SE = 0.203, *p* = 0.003), assisting patients to quit them by providing treatment or making a referral for treatment (Estimate = 0.613, SE = 0.252, *p* =0.016), and arranging to follow up with patients to assess their progress regarding quitting non-cigarette tobacco products (Estimate = 0.760, SE = 0.248, *p* = 0.002). By post-implementation, providers had higher odds of endorsing ask (OR: 1.836), assist (OR: 1.846), and arrange (OR: 2.137) relative to pre-implementation (Table 2).

### 3.3. The Effect of Comprehensive Tobacco-Free Workplace Implementation on Provider Beliefs, Provider Self-Efficacy, Receipt of Intervention Training, and Perceived Barriers to Treatment

Results from linear mixed models showed significant changes in provider beliefs (Estimate = 0.127, SE = 0.037, *p* = 0.001), provider self-efficacy (Estimate = 0.106, SE = 0.049, *p* = 0.032), receipt of intervention training (Estimate = 0.386, SE = 0.044, *p* < 0.001), and perceived barriers to treatment from pre- to post-implementation. By post-implementation, providers had higher odds of endorsing that it was as critical to treat tobacco use as it is to treat other substance use throughout treatment (i.e., provider beliefs; OR: 1.136), that they felt like they were able to deliver TUAs to patients effectively (i.e., self-efficacy; OR: 1.112), and that they received training on the use of counseling and behavioral therapies to treat tobacco use (i.e., intervention training receipt; OR: 1.471). Likewise, providers reported fewer barriers in regularly treating tobacco-using patients by post-implementation (Estimate = −0.551, *p* < 0.001) (Table 3).

### 3.4. The Effect of Center-Level Change on Provider Beliefs, Provider Self-Efficacy, Receipt of Intervention Training, and Perceived Barriers to Treatment on the Change of 5A’s for Non-Cigarette Tobacco Use 

#### 3.4.1. Provider Beliefs

The sample-dependent, median split, SUTC-level value differentiating low versus high changes over time in provider beliefs was 9.52. The moderating effect of the center-level change in provider beliefs predicted significant changes over time in advising non-cigarette tobacco users to quit (Estimate = 1.293, SE = 0.497, *p* = 0.010), assessing their interest in quitting (Estimate = 1.127, SE = 0.522, *p* = 0.032), assisting them with quitting through intervention or referral (Estimate = 1.687, SE = 0.520, *p* = 0.001), and arranging a follow-up (Estimate = 1.226, SE = 0.510, *p* = 0.017) (Table 4). 

Providers from centers with a larger percentage change in the belief that tobacco use should be addressed during substance use treatment had significantly greater odds of advising patients to quit non-cigarette tobacco use (OR from pre- to post-implementation: 1.179 to 3.001), assessing their interest in quitting (OR from pre- to post-implementation: 1.559 to 2.690), assisting with a quit attempt (OR from pre- to post-implementation: 0.562 to 2.390), and arranging a follow-up (OR from pre- to post-implementation: 0.328 to 1.328) over time. 

#### 3.4.2. Provider Self-Efficacy

The sample-dependent, median split, SUTC-level value differentiating low versus high changes over time in provider self-efficacy was 8.33. The center-level change in providers’ self-efficacy for the delivery of TUAs was not a significant moderator for changes in the delivery of any of the 5A’s from pre- to post-implementation (Table 4).

#### 3.4.3. Receipt of Intervention Training

The sample-dependent, median split, SUTC-level value differentiating low versus high changes over time in receipt of intervention training was 37.50. The center-level change in the receipt of intervention training was not a significant moderator for changes in the delivery of any of the 5A’s from pre- to post-implementation (Table 4).

#### 3.4.4. Provider Perceived Barriers

The sample-dependent, median split, SUTC-level value differentiating low versus high changes over time in providers’ perceived barriers was −0.58. The moderating effect of center-level change in providers’ perceived barriers to routinely providing TUD care predicted significant changes over time in advising non-cigarette tobacco users to quit (Estimate = 0.984, SE = 0.496, *p* = 0.048) and assisting them with quitting (Estimate = 1.403, SE = 0.518, *p* = 0.007) (Table 4).

Providers from centers with larger decreases in the percent of perceived barriers had significantly greater odds of endorsing that they advised non-cigarette tobacco users to quit (OR from pre- to post-implementation: 1.334 to 2.975) and assisted with patients’ quit attempts (OR from pre- to post-implementation: 0.563 to 2.123) over time. 

## 4. Discussion

A major purpose of this study was to examine how the education provided to SUTC providers through their center’s participation in a comprehensive tobacco-free workplace program affected intervention practices for patients’ non-cigarette tobacco use. Results indicated that non-cigarette tobacco intervention provision (i.e., the 5A’s) increased following program implementation, with significant increases in asking, assisting, and arranging. These results support that provider education delivered through tobacco-free workplace interventions can increase the provision of evidence-based care and extend prior work focused on the delivery of the 5A’s for conventional cigarettes [39,40,41,54,55] to non-cigarette tobacco use. Consequently, the implementation of similar programs at other SUTCs may be useful to address their patients’ non-cigarette tobacco product use, especially in geographical settings where use rates are elevated (e.g., rural counties [57]). 

While these results support that education can bolster providers’ non-cigarette intervention capacity, they also illuminate challenges in shifting rates of advising patients to quit and assessing patients’ willingness to make a quit attempt for non-cigarette tobacco products. One potential explanation for why increases in advising and assisting were not statistically significant is that they each had the highest baseline level of provider provision relative to the other A’s. Thus, providers who achieved changes in other intervention practices may have already been regularly advising patients to quit and assessing their willingness to quit. In those cases, education may have highlighted the importance of the other A’s and provided information that enabled their more routine practice. These results may suggest the need for provider education programs to emphasize the importance of assessing interest in quitting, particularly as a mechanism to build motivation for a quit attempt and honor patient autonomy. Similarly, increasing the practice of advising patients to quit (e.g., by providing information on non-cigarette forms of tobacco’s contribution to chronic disease) prior to asking patients about their interest in quitting should be emphasized during training [58]. Thus, more attention to the role and importance of assessing and advising may be needed in future program implementations to achieve significant increases in their use. 

Although providers’ intervention practices on non-cigarette tobacco use increased with education, they remained inconsistent with recommended practice to deliver the 5A’s at every patient visit [36]. Moreover, relative to the percentage of providers engaging in each of the 5A’s for cigarette smoking as indicated in prior work [40], a lower proportion of providers were doing the same for non-cigarette tobacco use. Together, results suggest that more efforts are needed to eliminate tobacco-related health disparities in these settings [14,15,16,28,59,60]. In addition to enhancing the focus on non-cigarette tobacco use in provider education sessions, another strategy to increase provider use of the 5A’s includes enacting a hard stop in electronic health records that prevents them from making further modifications to a healthcare record until performing these assessments [61]. Features of this mechanism could include prompts to focus on all tobacco product use. Anecdotally, however, the majority of the participating SUTCs used paper records or were unable to change their electronic health records without incurring significant expenses, prohibiting this option. Consequently, non-electronic provider nudge strategies (e.g., badge card reminders) [62] might be implemented and evaluated in future work as a mechanism to complement education provision and increase SUTC providers’ non-cigarette tobacco intervention practices. 

Social cognitive theory suggests that education may improve providers’ delivery of non-cigarette tobacco intervention by affecting increases in key factors (e.g., provider self-efficacy) that facilitate behavior change [49]; prior research supports the notion that training periods as short as 1 h can engender such changes [46]. In our sample of SUTCs, only about 39% of providers had received tobacco training in the year prior to program implementation; this number almost doubled by post-implementation. Low pre-implementation training receipt rates are consistent with those reported within prior studies in healthcare settings [63] and in SUTCs in particular [29]. However, consistent with expectations of the outcomes of increased training receipt, there were significant increases in provider beliefs that it was as critical to treat tobacco use as it was to treat other substance use throughout treatment and in provider self-efficacy to deliver TUAs effectively. Accordingly, barriers to the regular treatment of patients’ tobacco use declined. Therefore, provider education appears critical to equipping providers to treat non-cigarette tobacco dependency in SUTCs. Fortunately, there is a proliferation of resources in the U.S. that provide education on treating tobacco use, free of charge and conveniently via the internet (e.g., UCSF’s Smoking Cessation Leadership Center) [64]. SUTCs could reap the benefits of training by increasing providers’ access to tobacco-related education. The growing tobacco landscape in conjunction with a lack of training reported by SUTC providers are critical challenges for their understanding of the severity of non-cigarette tobacco use and acquiring skills for intervention [65,66]. Therefore, specific attention to ensuring non-cigarette tobacco use coverage in provider educational efforts is critical. 

The final contributions of this study to the literature are related to how changes in key provider constructs at the center level moderated providers’ non-cigarette intervention behaviors. These analyses recognize that employee behavior change within organizational settings is not only informed by individual factors but also by the broader ecological context of the inner setting [67,68,69]. Moreover, examining the effects of education receipt on provider care at the SUTC level is of interest given that SUTC practices can affect the tobacco use prevalence in an entire catchment area, particularly in more rural areas where access to care is sparse and tobacco-related health disparities may be more common [57]. Results indicated that SUTCs achieving high changes (increases) in provider beliefs about the importance of treating tobacco in SUTC care had significantly higher odds of advising, assessing, assisting, and arranging than centers with low changes in these provider beliefs. This may indicate that SUTCs for which exposure to these concepts is rather novel, or where employee beliefs largely did not prioritize tobacco intervention, have greater room to improve their non-cigarette tobacco use care provision. Results additionally indicated that SUTCs with the greatest reduction in provider-perceived barriers for intervention also demonstrated the greatest changes (increases) in providers intervening in non-cigarette tobacco use by advising and assisting patients to quit. These results may suggest that, for these centers, lack of knowledge was a primary deterrent to patient care for non-cigarette tobacco use. Alternatively, there may be other unknown or unmeasured factors (e.g., evident leadership support, adequacy of staffing for caseload) that distinguished some SUTCs from others regarding barriers to care provision and their ability to change over time. Nonetheless, these findings demonstrate that SUTCs that achieve the greatest reduction in perceived barriers and improvements in the prioritization of tobacco treatment are likely to exhibit the largest improvements in non-cigarette tobacco intervention from tobacco-free workplace program implementation. 

Although this tobacco-free workplace program implementation bolstered SUTCs’ intervention practices for non-cigarette tobacco use, more work is needed. Specifically, the extent to which the brief education session—one of several components of the overall program—was responsible for findings is not definitively known. Future work to tease apart the effects of each core component on provider behavior could contribute to more targeted interventions and identify parts of the program that could benefit from greater development. Moreover, it is important to note that the extent to which these gains are sustainable and cost-effective over longer periods of time was not assessed in the current program and should be a focus of future work. Notably, SUTCs are known for high provider turnover [53]; thus, continuing education and other center-level sustainment strategies may be necessary to preserve an inner SUTC context that values the extension of substance use disorder treatment to non-cigarette TUD intervention [44,45]. Prior work suggests that a champion-led train-the-trainer program may facilitate the sustainment of educational efforts and TUD care provision in behavioral health settings [70,71]; however, results require replication in SUTC settings specifically. Lastly, greater center-level changes in providers’ receipt of training and self-efficacy for TUAs seemed less important to center shifts in non-cigarette tobacco care provision than other center-level provider factors, providing opportunities for future replication and further investigation. 

A clear strength of this study was its longitudinal design and potential to assess causational relationships; however, there are several weaknesses in addition to those already noted that bear mention. These include that provider survey responses were anonymous and could not be matched from pre- and post-implementation. This made it impossible to assess individual-provider-level constructs and their impact on intervention uptake as well as the impact of potentially variable turnover rates between SUTCs. Future studies should collect information on the total number of providers exactly versus in ranges, and at both the pre- and post-implementation time points so that response rates can be calculated. The current study also saw lower participation in post- than pre-implementation surveys, perhaps affected by provider fatigue. The current study was not funded to include remuneration for survey completion, and some state-supported treatment centers may disallow employees to accept compensation for participation during regular working hours. Nevertheless, there may be opportunities to engage SUTC leadership to promote post-implementation survey completion that were not pursued in this study. In addition, provider intervention behaviors were self-reported and subject to personal bias. Although anonymous data collection was meant to facilitate honest responses, it is unknown whether this was fully achieved. Future work may include provider documentation/chart reviews and data collection at the patient level as well as supplement provider self-reports.

## 5. Conclusions

Results from the current study demonstrate that tobacco-free workplace program implementation with provider education may be an effective strategy to address SUTC patients’ non-cigarette tobacco product use by bolstering SUTC providers’ non-cigarette tobacco intervention capacity. Although TTTF had several core components, prior research suggests that provider education may be key to changing provider non-cigarette tobacco care provision [46,47]. Indeed, TTTF education was designed to affect theory-based provider factors that impact behavioral change, and changes were seen in each of these constructs. However, more attention to the effects of specific implementation strategies on maximizing provider behavior change is needed in future work, as is attention to how implementation-associated costs and provider turnover may affect program adoption and sustainment. Nevertheless, the current work highlights the promise of tobacco-free workplace programs (with provider educational components) in improving care for non-cigarette tobacco dependency for patients in substance use disorder treatment, expanding the literature beyond attention to cigarette smoking to other increasingly prevalent forms of tobacco use that harm patient health. Still, more work is needed to ensure that SUTC providers treat both cigarette and non-cigarette tobacco use more routinely. Additional work in this area is vital to mitigating the tobacco-related health inequities experienced by patients in treatment for non-tobacco substance use. 

## Figures and Tables

**Table 1:** Providers’ Delivery of the 5A’s for Non-cigarette Tobacco Use From Before to After a Comprehensive Tobacco-Free Workplace Program Implementation Within Each of the 15 Participating Substance Use Treatment Centers.

	Ask		*p*-Value	Advise		*p*-Value	Assess		*p*-Value	Assist		*p*-Value	Arrange		*p*-Value
	Pre (%)	Post (%)		Pre (%)	Post (%)		Pre (%)	Post (%)		Pre (%)	Post (%)		Pre (%)	Post (%)	
All SUTCs	50.97	64.43	**0.004**	56.72	60.12	0.549	67.67	65.34	0.668	48.87	58.62	0.089	39.10	53.45	**0.013**
SUTC1	64.00	68.18	0.763	50.00	80.00	0.135	78.57	70.00	0.704	57.14	85.00	0.116	50.00	55.00	0.774
SUTC2	66.67	100.00	1.000	66.67	100.00	1.000	66.67	100.00	1.000	66.67	100.00	1.000	66.67	100.00	1.000
SUTC3	100.00	100.00	NA	50.00	100.00	0.200	66.67	100.00	0.467	33.33	100.00	0.076	33.33	75.00	0.524
SUTC4	54.55	92.31	0.061	66.67	90.00	0.303	55.56	80.00	0.350	44.44	50.00	1.000	44.44	60.00	0.656
SUTC5	66.67	100.00	0.429	50.00	75.00	1.000	100.00	75.00	1.000	0.00	50.00	0.467	50.00	75.00	1.000
SUTC6	42.00	52.50	0.321	38.10	45.95	0.562	42.86	52.78	0.470	9.52	43.24	**0.008**	0.00	37.84	**0.001**
SUTC7	60.87	68.42	0.611	60.00	22.22	0.170	90.00	53.85	0.089	50.00	30.00	0.650	40.00	40.00	1.000
SUTC8	33.33	50.00	1.000	66.67	100.00	1.000	33.33	100.00	0.400	66.67	100.00	1.000	33.33	100.00	0.400
SUTC9	32.31	46.00	0.134	47.83	41.67	0.624	54.55	54.17	0.976	45.45	47.92	0.848	36.36	47.92	0.366
SUTC10	75.00	100.00	1.000	80.00	100.00	1.000	100.00	100.00	NA	60.00	100.00	1.000	80.00	100.00	1.000
SUTC11	100.00	80.00	1.000	100.00	80.00	1.000	100.00	80.00	1.000	100.00	60.00	0.464	100.00	80.00	1.000
SUTC12	70.00	71.43	1.000	76.47	69.23	0.698	100.00	76.92	0.070	100.00	76.92	0.070	76.47	61.54	0.443
SUTC13	55.56	71.43	0.633	60.00	85.71	0.523	83.33	100.00	0.462	60.00	85.71	0.523	0.00	71.43	**0.028**
SUTC14	36.36	100.00	0.192	33.33	66.67	0.524	16.67	66.67	0.226	16.67	66.67	0.226	16.67	33.33	1.000
SUTC15	44.44	87.50	0.131	71.43	87.50	0.569	66.67	75.00	1.000	42.86	75.00	0.315	28.57	75.00	0.132

Note. Chi-square tests/Fisher exact tests were conducted, as appropriate, to examine pre- to post-implementation changes in the 5A’s. SUTC = substance use treatment center. The number of providers completing pre-implementation surveys within centers ranged from 3 to 65 (total pre-implementation n = 259 for all centers combined). The number of providers completing post-implementation surveys within centers ranged from 1 to 50 (total post-implementation n = 194 for all centers combined). Bolded values represent significance at *p* < 0.05.

**Table 2:** Changes in Providers’ Delivery of the 5A’s for Non-cigarette Tobacco Use From Before to After a Comprehensive Tobacco-Free Workplace Program Implementation (N = 15 SUTCs).

	Time (Ref: Pre-Implementation)		
	Estimate	SE	OR (95% CI)
Ask	0.608	0.203	1.836 (1.234, 2.733)
Advise	0.292	0.245	1.339 (0.828, 2.165)
Assess	0.034	0.255	1.035 (0.628, 1.706)
Assist	0.613	0.252	1.846 (1.126, 3.026)
Arrange	0.760	0.248	2.137 (1.314, 3.477)

Note. SE: Standard Error; OR: Odds Ratio; SUTC: substance use treatment center; CI: Confidence Interval.

**Table 3:** The Effect of Comprehensive Tobacco-Free Workplace Program Implementation on Provider Beliefs, Provider Self-Efficacy, Receipt of Intervention Training, and Perceived Barriers to Treatment (N = 15 SUTCs).

Outcome	Time (Ref: Pre-Implementation)		
	Estimate	SE	OR (95% CI)/*p*-Value
Provider beliefs	0.127	0.037	1.136 (1.057, 1.221)
Provider self-efficacy *	0.106	0.049	1.112 (1.009, 1.225)
Receipt of intervention training	0.386	0.044	1.471 (1.349, 1.605)
Perceived barriers to treatment	−0.551	0.105	<0.001

Note. SE: Standard Error; OR: Odds Ratio; SUTC: substance use treatment center; CI: Confidence Interval; * n = 11 centers.

**Table 4:** Center-level Changes as Moderators of Providers’ Delivery of the 5A’s for Non-cigarette Tobacco Use from Pre- to Post-Implementation of a Comprehensive Tobacco-Free Workplace Program (N = 15 SUTCs).

Provider Behaviors	Effect	Estimate	SE	*p*-Value
Ask	Time (ref: pre-implementation)	0.463	0.283	0.102
	Provider beliefs (ref: low beliefs change)	−0.143	0.453	0.753
	Interaction Term	0.300	0.407	0.462
	Time (ref: pre-implementation)	0.401	0.463	0.387
	Provider self-efficacy (ref: low self-efficacy change) *	−0.786	0.492	0.111
	Interaction Term	0.329	0.532	0.536
	Time (ref: pre-implementation)	0.634	0.266	0.018
	Receipt of intervention training (ref: low change in receiving training)	0.546	0.440	0.216
	Interaction Term	−0.050	0.412	0.904
	Time (ref: pre-implementation)	0.424	0.279	0.130
	Perceived barriers to treatment (ref: decrease fewer or increase barriers)	−0.360	0.458	0.432
	Interaction Term	0.390	0.408	0.339
Advise	Time (ref: pre-implementation)	−0.358	0.352	0.309
	Provider beliefs (ref: low beliefs change)	−0.651	0.508	0.201
	Interaction Term	1.293	0.497	0.010
	Time (ref: pre-implementation)	1.076	0.563	0.057
	Provider self-efficacy (ref: low self-efficacy change) *	−0.276	0.608	0.650
	Interaction Term	−0.892	0.652	0.173
	Time (ref: pre-implementation)	0.152	0.330	0.645
	Receipt of intervention training (ref: low change in receiving training)	0.102	0.503	0.840
	Interaction Term	0.327	0.496	0.511
	Time (ref: pre-implementation)	−0.182	0.344	0.598
	Perceived barriers to treatment (ref: decrease fewer or increase barriers)	−0.396	0.510	0.439
	Interaction Term	0.984	0.496	0.048
Assess	Time (ref: pre-implementation)	−0.582	0.388	0.135
	Provider beliefs (ref: low beliefs change)	−1.083	0.555	0.052
	Interaction Term	1.127	0.522	0.032
	Time (ref: pre-implementation)	−0.135	0.602	0.823
	Provider self-efficacy (ref: low self-efficacy change) *	−1.371	0.551	0.014
	Interaction Term	0.406	0.685	0.554
	Time (ref: pre-implementation)	−0.050	0.337	0.882
	Receipt of intervention training (ref: low change in receiving training)	0.531	0.554	0.339
	Interaction Term	0.226	0.520	0.665
	Time (ref: pre-implementation)	−0.415	0.381	0.277
	Perceived barriers to treatment (ref: decrease fewer or increase barriers)	−1.044	0.544	0.056
	Interaction Term	0.827	0.518	0.112
Assist	Time (ref: pre-implementation)	−0.240	0.363	0.509
	Provider beliefs (ref: low beliefs change)	−1.280	0.576	0.027
	Interaction Term	1.687	0.520	0.001
	Time (ref: pre-implementation)	0.989	0.539	0.068
	Provider self-efficacy (ref: low self-efficacy change) *	−0.898	0.520	0.086
	Interaction Term	−0.305	0.635	0.631
	Time (ref: pre-implementation)	0.437	0.333	0.190
	Receipt of intervention training (ref: low change in receiving training)	−0.219	0.587	0.710
	Interaction Term	0.437	0.517	0.399
	Time (ref: pre-implementation)	−0.076	0.357	0.831
	Perceived barriers to treatment (ref: decrease fewer or increase barriers)	−1.298	0.579	0.026
	Interaction Term	1.403	0.518	0.007
Arrange	Time (ref: pre-implementation)	0.172	0.348	0.622
	Provider beliefs (ref: low beliefs change)	−1.532	0.508	0.003
	Interaction Term	1.226	0.510	0.017
	Time (ref: pre-implementation)	0.790	0.504	0.119
	Provider self-efficacy (ref: low self-efficacy change) *	−0.984	0.632	0.121
	Interaction Term	0.327	0.622	0.600
	Time (ref: pre-implementation)	0.555	0.329	0.092
	Receipt of intervention training (ref: low change in receiving training)	−0.243	0.545	0.656
	Interaction Term	0.511	0.509	0.316
	Time (ref: pre-implementation)	0.413	0.342	0.228
	Perceived barriers to treatment (ref: decrease fewer or increase barriers)	−0.720	0.530	0.175
	Interaction Term	0.746	0.503	0.139

Note. SE: Standard Error; SUTC: substance use treatment center; * n = 11 centers.

## Data Availability

The data presented in this study are available on request from the corresponding author. The data are not publicly available due to confidentiality/privacy agreements with funders.
